# *Sox9* in the epicardium: Implications for cell invasion, differentiation, and coronary vascular development

**DOI:** 10.1371/journal.pone.0325852

**Published:** 2025-06-23

**Authors:** Andrew B. Harvey, Allison M. Trouten, Renélyn A. Wolters, Jenna R. Drummond, Raymond N. Deepe, Hannah G. Tarolli, Inara Devji, Silvia G. Vaena, Martin J. Romeo, Robin Muise-Helmericks, Paula S. Ramos, Russell A. Norris, Ge Tao, Andy Wessels

**Affiliations:** 1 Department of Regenerative Medicine and Cell Biology, College of Medicine, Medical University of South Carolina, Charleston, South Carolina, United States of America; 2 Hollings Cancer Center, Medical University of South Carolina, Charleston, South Carolina, United States of America; 3 Department of Medicine, Medical University of South Carolina, Charleston, South Carolina, United States of America; Victor Chang Cardiac Research Institute, AUSTRALIA

## Abstract

The epicardium is the mesothelial lining of the heart and is a source of progenitor cells during heart development, giving rise to an invasive population of mesenchymal cells which differentiate into cardiac fibroblasts, mural cells, and other cell types essential for heart structure and function. Previously, we showed that epicardial-specific deletion of the gene encoding SRY-box transcription factor 9 (SOX9) impairs epicardial-derived cell invasion and reduces their contribution to the atrioventricular valve mesenchyme. In this study, we use single-cell RNA-sequencing to investigate broader roles of *Sox9* in the epicardium as it relates to epicardial invasion, differentiation, and vascular development. We identified transcriptional changes indicative of decreased epicardial-to-mesenchymal transformation consistent with histological observations. Immunofluorescence analyses revealed defective epicardial attachment and decreased epicardial-derived cell invasion into the ventricular myocardium associated with delayed coronary plexus formation. *Sox9*-deficient epicardial cells exhibited elevated expression of vascular smooth muscle cell genes, suggesting that *Sox9* may influence epicardial cell fate decisions. This study expands our understanding of the role of *Sox9* in epicardial biology, demonstrating an important function in regulating epicardial cell invasion, differentiation, and coronary vasculature development. These insights provide a foundation for further investigations into epicardial-mediated mechanisms underlying congenital heart abnormalities.

## Introduction

The epicardium acts as an important source of progenitor cells which give rise to multiple cell types essential for heart development. The proepicardial organ (PEO), the extracardiac precursor to the epicardium, is a coelomic mesothelium-derived cluster of cells situated dorsal to the developing heart [1–5]. Around embryonic day (E)9.5 in mouse, cells from the PEO migrate towards the surface of the heart where they spread across the myocardium to form a thin, continuous layer of epicardial cells [1]. A portion of these cells will subsequently undergo an epicardial-to-mesenchymal transformation (EpiMT) generating an invasive population of epicardial-derived cells (EPDCs). Around E12.5, EPDCs invade the myocardium and proceed to differentiate into multiple different cell types: predominantly cardiac fibroblasts and mural cells (vascular smooth muscle cells [VSMCs] and pericytes), but also, to a lesser extent, endothelial cells of the coronary vasculature [6–9]. We have previously reported on a subpopulation of EPDCs at the atrioventricular (AV) junction that contribute to the mesenchyme of the AV valves, where they play an important role in maintaining extracellular matrix homeostasis [10–13]. The epicardium and EPDCs act as a source of signals which modulate the growth and maturation of the myocardium and patterning of the coronary vasculature [14–16]. Despite significant advances in understanding the cell types derived from the epicardium, the timing and molecular mechanisms governing their migration and differentiation remain poorly understood.

SRY-Box Transcription Factor 9 (SOX9), is a high mobility group domain transcription factor with critical roles in embryonic development. While homozygous null mutations in *Sox9* are embryonic lethal [17], cell-type specific deletion of *Sox9* using the Cre-LoxP system has provided insight into its diverse roles in cardiovascular development. Deletion of *Sox9* from the endocardial or cardiac neural crest cell lineages results in defective cardiac cushion formation, the precursors to the valves [18,19]. Additionally, deletion of *Sox9* from the second heart field results in a high penetrance of ventricular septal defects, often in combination with primary atrial septal defects [20,21]. Recently, we identified a role for *Sox9* in the epicardium, showing that epicardial-specific deletion of *Sox9* leads to a reduced contribution of EPDCs to the AV valves and reduced EPDC invasion into the ventricular myocardium [13]. In that study, we focused mainly on the tissues of the AV junction, particularly the mitral valve. We reported that reduced contribution of EPDCs to the posterior leaflet of the mitral valve during development due to loss of *Sox9* leads to myxomatous valve degeneration postnatally [13]. Our previous study demonstrated that the presence of EPDCs in the valves is essential for maintaining valve extracellular matrix homeostasis [13].

Here, we used single-cell RNA-sequencing to further characterize the role of *Sox9* in epicardial development. Transcriptomic analyses of E14.5 epicardial-*Sox9* knockout and control hearts indicated dysregulation of genes implicated in epicardial invasion, differentiation, and vascular development. Immunofluorescence analyses revealed defective epicardial attachment and delay in coronary plexus formation associated with decreased EPDC invasion in epicardial-*Sox9* knockout hearts. *Sox9*-deficient epicardial cells expressed higher levels of VSMC genes suggestive of a role for *Sox9* in epicardial cell fate decisions. Our data provide insight into the role of *Sox9* in coordinating epicardial cell behaviors important for proper heart development.

## Results

### SOX9 regulates epicardial-derived cell invasion during heart development

**Fig 1:**
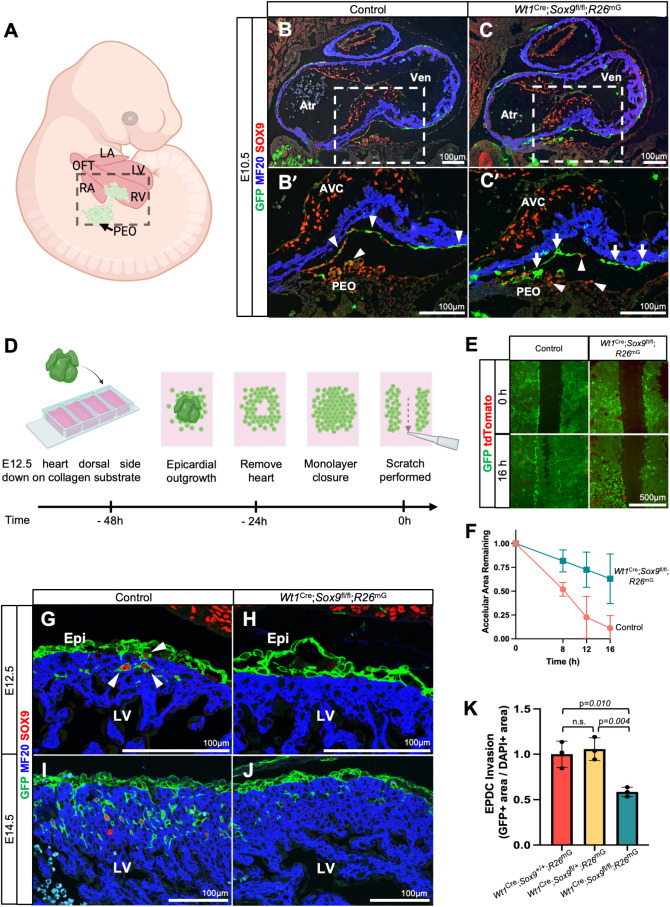
SOX9 regulates epicardial-derived cell invasion. **(A)** Schematic showing epicardial formation from the proepicardial organ. Boxed region indicates area shown in (B–C’). (B–C’) Immunofluorescence shows *in vivo* SOX9 expression (red) in control (B, B’) and *Wt1*^Cre^; *Sox9*^fl/fl^; *R26*^mG^ (C, C’) embryonic hearts at E10.5. MF20 (blue) is used as a myocardial marker. The *Wt1*^Cre^ lineage is marked by GFP (green). Arrowheads in (B’) highlight SOX9 + epicardial cells as they migrate from the proepicardial organ (PEO) to envelop the myocardium. Arrows in (C’) indicate epicardial cells which have undergone Cre-mediated recombination and no longer express SOX9. **(D)** Schematic of epicardial explant and scratch assay. (E, F) Fluorescence images of epicardial explants from control and *Wt1*^Cre^; *Sox9*^fl/fl^; *R26*^mG^ hearts at t = 0 and t = 16 hours post scratch (E), quantified in (F) (n = 2). Datapoints represent mean ±s.d. **(G–J)** SOX9 expression in control (G, I) and *Wt1*^Cre^; *Sox9*^fl/fl^; *R26*^mG^ (H, J) embryonic hearts at E12.5 (G, H) and E14.5 **(I, J)**. By E12.5, SOX9 is no longer prominently expressed in the epicardium proper, but it is upregulated in a subset of EPDCs as they undergo EpiMT and invade the underlying ventricular myocardium (arrowheads, **(G)**). **(K)** *Wt1*^Cre^; *Sox9*^fl/fl^; *R26*^mG^ specimens display reduced EPDC invasion in comparison to both homozygous wildtype and heterozygous specimens (*Wt1*^Cre^; *Sox9*^+/+^; *R26*^mG^ and *Wt1*^Cre^; *Sox9*^fl/+^; *R26*^mG^). Datapoints are mean ± s.d. (n = 3). (Control hearts are from littermate matched *Wt1*^Cre^; *Sox9*^fl/+^; *R26*^mG^ specimens. Atr, atrium; AVC, atrioventricular cushion; Epi, epicardium; LV, left ventricle; PEO, proepicardial organ; Ven, ventricle).

Cells from the proepicardial organ (PEO), the extracardiac precursor to the epicardium, migrate towards and envelop the developing mouse heart, beginning around E9.5 [1] (Fig 1A). In our previous study, we described the generation of epicardial-specific *Sox9* knockout mice using the previously described Wt1-Cre mouse (*Wt1*^Cre^) [11,13]. By crossing in a *Rosa26*^mTmG^ reporter allele (abbreviated *R26*^mG^), we were able to trace the epicardium and its progeny by GFP expression detected by immunofluorescence. For all histological and immunofluorescence analyses, mice heterozygous at the *Sox9* locus (*Wt1*^Cre^; *Sox9*^fl/+^; *R26*^mG^) were phenotypically indistinguishable from *Wt1*^Cre^; *Sox9*^+/+^; *R26*^mG^ specimens across all measured parameters, including epicardial attachment, EPDC invasion, myocardial thickness, and valve morphology [13]. They were thus deemed appropriate controls for comparison to *Wt1*^Cre^; *Sox9*^fl/fl^; *R26*^mG^ littermates. This was consistent with prior studies using *Sox9* conditional alleles in related contexts [13,20,21].

We examined SOX9 expression in control and *Wt1*^Cre^; *Sox9*^fl/fl^; *R26*^mG^ hearts by immunofluorescence in the early stages of the establishment of the epicardium. At E10.5, SOX9 was robustly expressed in the PEO, in epicardial cells as they migrate over the surface of the myocardium, and in the AV cushions of control specimens (Fig 1B, B’, arrowheads). In *Wt1*^Cre^; *Sox9*^fl/fl^; *R26*^mG^ specimens, SOX9 was absent in GFP-labeled cells that had undergone Cre-mediated recombination in both the PEO and epicardium (Fig 1C, C’, arrows). Importantly, however, SOX9 was still present in GFP-negative populations of the PEO and epicardium at this stage, suggesting incomplete recombination by the *Wt1*^Cre^ at this timepoint. Notably, the early establishment of the epicardium appeared grossly normal in *Wt1*^Cre^; *Sox9*^fl/fl^; *R26*^mG^ specimens, though incomplete recombination at this stage precludes the conclusion that *Sox9* does not play a role in this process.

After epicardial cells cover the surface of the heart, a subset will undergo an epicardial-to-mesenchymal transformation (EpiMT), giving rise to epicardial-derived cells (EPDCs), which will migrate into the underlying myocardium [6,8,22,23]. To determine if SOX9 affects the migratory capacity of epicardial cells, we performed a scratch closure migration assay on epicardial explants from control (*Wt1*^Cre^; *Sox9*^fl/+^; *R26*^mG^) and *Wt1*^Cre^; *Sox9*^fl/fl^; *R26*^mG^ hearts (Fig 1D). By E12.5, all epicardial cells expressed GFP, indicating effective Cre-mediated recombination. Fluorescence imaging of explant cultures confirmed low non-epicardial cell contamination (Fig 1E). Epicardial cells explanted from *Wt1*^Cre^; *Sox9*^fl/fl^; *R26*^mG^ hearts showed diminished ability to migrate into the acellular area after scratch when compared to controls (Fig 1E, 1F).

To examine EPDC invasion *in vivo*, we examined hearts by immunofluorescence at E12.5 and E14.5. At E12.5, SOX9 expression was negligible in the epicardium proper, but became upregulated in EPDCs undergoing epicardial-to-mesenchymal transformation (EpiMT) as they invaded the ventricular myocardium (Fig 1G, arrowheads). In *Wt1*^Cre^; *Sox9*^fl/fl^; *R26*^mG^ hearts, the loss of *Sox9* results in diminished EPDC invasion into the myocardium (Fig 1H). At E14.5, control hearts displayed extensive EPDC invasion into the myocardium, with heterogeneous expression of SOX9 (Fig 1I). In contrast, *Wt1*^Cre^; *Sox9*^fl/fl^; *R26*^mG^ hearts exhibited a marked reduction of EPDC infiltration (Fig 1J). The reduction in EPDC invasion at E14.5 was quantified to be approximately 40% in *Wt1*^Cre^; *Sox9*^fl/fl^; *R26*^mG^ hearts compared to both *Wt1*^Cre^; *Sox9*^+/+^; *R26*^mG^ and *Wt1*^Cre^; *Sox9*^fl/+^; *R26*^mG^ specimens (Fig 1K). We previously reported that reduced EPDC invasion was also accompanied by the presence of a thinner compact myocardial wall and decreased cardiomyocyte proliferation [13]. Overall, these findings suggest that *Sox9* plays an important role in the migratory capacity of EPDCs as they transform to an invasive mesenchymal state.

### Single-cell RNA-sequencing of E14.5 hearts

**Fig 2:**
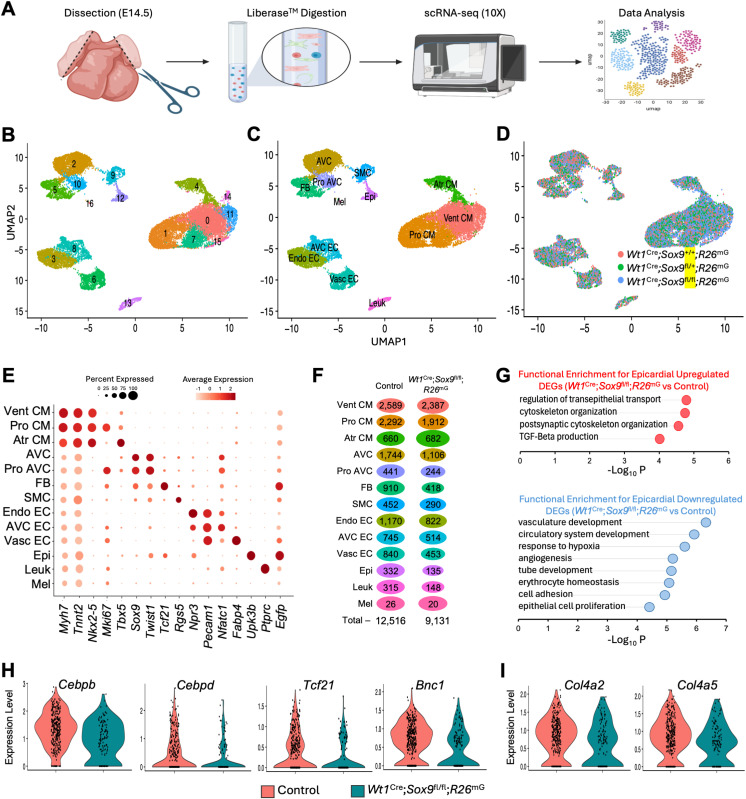
Single-cell RNA-sequencing of E14.5 hearts. **(A)** Schematic showing the scRNA-seq sample preparation and experimental workflow. **(B)** Uniform Manifold Approximation Projection (UMAP) representation of unbiased clustering analysis of 21,918 single-cell transcriptomes identifying 17 transcriptionally distinct clusters. **(C)** Final annotated clusters after exclusion of low-quality clusters (14 and 15) and merging of similar cardiomyocyte clusters (1 and 7, 0 and 11). **(D)** UMAP colored by genotype. **(E)** Dot plot of gene expression for representative marker genes (x-axis) for each cell type cluster (y-axis) represented in **(C)**. **(F)** Frequency plot depicting number of cells in each cluster, split by group, with the size of the bubble indicating the proportion of the total number of cells in the group contained in that cluster, to normalized to the size of the control group. **(G)** Biological Processes from functional enrichment analysis of significantly upregulated and downregulated genes (p_adj_ < 0.05, |FC| > 1.25) in *Wt1*^Cre^; *Sox9*^fl/fl^; *R26*^mG^ epicardial cells compared to controls. (H,I) Violin plots indicating relative expression levels of a subset of differentially expressed genes in control and *Wt1*^Cre^; *Sox9*^fl/fl^; *R26*^mG^ epicardial cells. These genes have been previously implicated in important epicardial processes such as EpiMT, EPDC differentiation, and epicardial attachment. (Atr CM, atrial cardiomyocytes; AVC, atrioventricular cushion; AVC EC, atrioventricular cushion endothelial cells; Endo EC, endocardial endothelial cells; Epi, epicardium; FB, fibroblasts; Leuk, leukocytes; Mel, melanocytes; Pro AVC, proliferating atrioventricular cushion; Pro CM, proliferating cardiomyocytes; SMC, smooth muscle cells; Vasc EC, vascular endothelial cells; Vent CM, ventricular cardiomyocytes).

To better understand how loss of *Sox9* in the epicardium affects heart development, we employed single-cell RNA-sequencing (scRNA-seq) to profile the transcriptome from *Wt1*^Cre^; *Sox9*^+/+^; *R26*^mG^ (n = 3 pooled), *Wt1*^Cre^; *Sox9*^fl/+^; *R26*^mG^(n = 1), and *Wt1*^Cre^; *Sox9*^fl/fl^; *R26*^mG^ (n = 3 pooled) hearts at E14.5. To enrich for cell populations of interest, we removed most of the left and right atrial appendage during microdissection (Fig 2A). Unbiased clustering visualized through a uniform manifold approximation and projection plot (UMAP) identified 17 transcriptionally distinct clusters (Fig 2B). After quality control, we obtained a dataset of 21,647 single cell transcriptomes across the three genotypes. Clusters were further merged based upon expression of known cell type markers into 13 clusters (Fig 2C). Cells from each genotype were present in each of the 13 clusters, which ranged in size from 46 cells to 4,976 cells (Fig 2D, S1 Fig). Given the absence of an observable phenotype in *Wt1*^Cre^; *Sox9*^fl/+^; *R26*^mG^ specimens, *Wt1*^Cre^; *Sox9*^+/+^; *R26*^mG^ and *Wt1*^Cre^; *Sox9*^fl/+^; *R26*^mG^ data were grouped together as controls in downstream differential expression analyses for comparison to *Wt1*^Cre^; *Sox9*^fl/fl^; *R26*^mG^to prioritize discovery genes associated with the knockout phenotypes.

We performed differential expression analysis for each cluster compared to all other clusters to manually annotate cell types (S1 Table). A subset of the markers used to identify clusters in Fig 2C are represented in a dot plot in Fig 2E. Epicardial (Epi) cells were identified by expression of the mesothelial and epicardial markers *Upk3b*, *Wt1*, and *Tbx18* [11,24,25]. Fibroblasts (FB) were identified by high expression of *Tcf21*, *Spon2*, and *Col1a1* [26]. Additionally, detection of the *Rosa26*^mTmG^ reporter transcript, enhanced green fluorescent protein (*Egfp*), allowed for identification of the epicardial lineage, notably highly expressed in the Epi and FB clusters. The smooth muscle cell (SMC) cluster, marked by expression of *Rgs5*, *Cspg4*, and *Cxcl12* [27] was largely *Egfp*-negative, indicating that this is likely a cluster of outflow tract SMCs of cardiac neural crest and second heart field origin. A subset of cells within the FB cluster, which are *Egfp+*, *Rgs5*+, *Kcnj8+*, and *Cspg4*+, likely represent mural cells of epicardial origin [28]. A full list of cell type markers can be found in S1 Table. Feature plots of a subset of these markers overlayed on the UMAP plot are displayed in S1 Fig.

Comparison of cell type proportions between control and *Wt1*^Cre^; *Sox9*^fl/fl^; *R26*^mG^ hearts revealed differences in cellular composition between groups (Fig 2F, S1 Fig). Notably, the Epi, FB, and Vasc EC clusters were substantially reduced in *Wt1*^Cre^; *Sox9*^fl/fl^; *R26*^mG^ compared to controls, while the proportions of cardiomyocyte (CM) clusters were reciprocally increased (Fig 2F, S1 Fig). The reduced proportion of epicardial cells suggests that loss of *Sox9* may impair epicardial survival or proliferation, leading to a smaller epicardial population, although we did not observe gross abnormalities in the epicardium proper at E14.5 (Fig 1J). Given that fibroblasts arise from the epicardium following EpiMT, a nearly 40% proportional decrease in the FB cluster in *Wt1*^Cre^; *Sox9*^fl/fl^; *R26*^mG^ compared to controls further supports a reduction in EPDC presence in *Wt1*^Cre^; *Sox9*^fl/fl^; *R26*^mG^ hearts, reduced in a similar proportion in quantifications from immunofluorescent stains (Fig 1K). While vascular endothelial cells are not predominantly derived from the epicardium [29], their reduction is likely an indirect consequence of diminished EPDCs in the myocardial wall. The epicardium and EPDCs contribute signals that regulate vascularization in a paracrine manner [9,30,31]. Thus, the loss of these supportive cues in *Wt1*^Cre^; *Sox9*^fl/fl^; *R26*^mG^ hearts may underlie the observed decrease in Vasc EC. These findings suggest that *Sox9* is essential for maintaining epicardial integrity, with broad consequences for downstream mesenchymal and endothelial lineages.

### Dysregulation of genes implicated in epicardial invasion and differentiation

We next performed differential expression analysis between the epicardial cluster in control and *Wt1*^Cre^; *Sox9*^fl/fl^; *R26*^mG^ specimens (S2 Table). We input differentially expressed upregulated and downregulated genes (p_adj_ < 0.05, Log2 |FC| > 1.25) to ToppGene suite’s functional enrichment analysis tool, ToppFun [32], which revealed upregulation of genes in cytoskeletal and epithelial-barrier related pathways in *Wt1*^Cre^; *Sox9*^fl/fl^; *R26*^mG^ epicardial cells compared to controls (Fig 2G). Genes that were downregulated were enriched for biological processes related to vasculature development and angiogenesis (Fig 2G).

Downregulated genes included various transcriptional regulators, secreted factors, and ECM components that have been previously implicated in a variety of processes in epicardial biology. *Wt1*^Cre^; *Sox9*^fl/fl^; *R26*^mG^ epicardial cells displayed dysregulation of factors known to influence EpiMT including *Cebpb, Cebpd*, and *Tcf21* [33,34] (Fig 2H). While TCF21 and C/EBP-family transcription factors have been shown to be positive regulators of EpiMT that were downregulated in *Wt1*^Cre^; *Sox9*^fl/fl^; *R26*^mG^ epicardial cells, we also saw upregulation of *Cldn3* and *Crip1,* negative regulators of EMT [35,36] (S2 Fig). Additionally, we observed downregulation of two transcription factors reported to influence epicardial heterogeneity and differentiation potential, *Bnc1* and *Tcf21* [34,37–39] (Fig 2H). Epicardial cells from *Wt1*^Cre^; *Sox9*^fl/fl^; *R26*^mG^ hearts expressed lower levels of secreted factors known to influence myocardial growth when compared to controls, including cardiomyocyte mitogens *Fgf2* and *Fgf9* [40,41], and canonical Wnt signaling ligand *Wnt5a* [42], offering a potential explanation for decreased cardiomyocyte proliferation described in this model [13] (S2 Fig). Overall, these data support a model in which the loss of *Sox9* results in a transcriptional program rendering the epicardium less conducive to undergoing EpiMT, supporting our immunofluorescence observations of reduced numbers of EPDCs within the myocardial wall at this timepoint (Fig 1). Additionally, we identified downregulation of two type IV collagen chains, *Col4a2* and *Col4a5*, in *Wt1*^Cre^; *Sox9*^fl/fl^; *R26*^mG^ epicardial cells when compared to controls (Fig 2I).

### SOX9 mediates epicardial attachment to underlying myocardium

**Fig 3:**
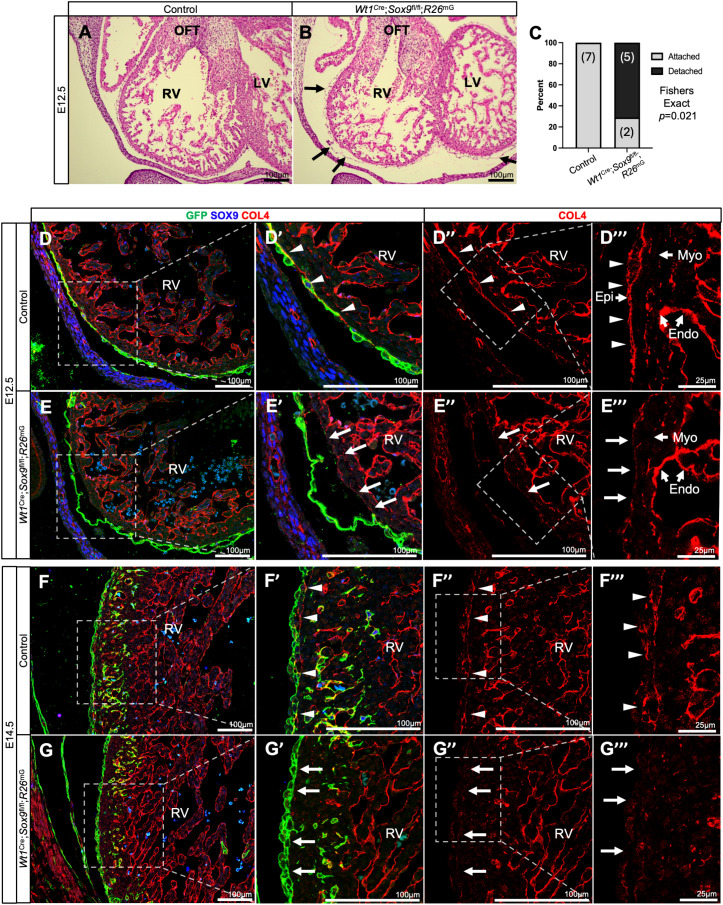
Loss of *Sox9* results in defective epicardial attachment. **(A–C)** Hematoxylin and Eosin stains show control (A) and *Wt1*^Cre^; *Sox9*^fl/fl^; *R26*^mG^ (B) hearts at E12.5. While the epicardium was adhered to the myocardium in all control specimens examined at this time point, arrows in (B) highlight epicardial blistering seen in 5/7 *Wt1*^Cre^; *Sox9*^fl/fl^; *R26*^mG^ specimens as quantified in **(C)** (Fisher’s Exact Test). (D–E”’) Immunofluorescent stains show the epicardium (GFP, green) and the basement membrane type 4 collagen (COL4, red) in control (D–D”’) and *Wt1*^Cre^; *Sox9*^fl/fl^; *R26*^mG^ (E–E”’) embryonic hearts at E12.5. Arrowheads in (D’–D”’) highlight the epicardium anchored to the underlying myocardium (Myo) by a continuous COL4 + basement membrane, while arrows in (E’–E”’) indicate lower COL4 expression in regions of epicardial blistering. (F–G”’) Immunofluorescent stains at E14.5 showing the epicardium and COL4 in control (F–F”’) and *Wt1*^Cre^; *Sox9*^fl/fl^; *R26*^mG^ (G–G”’) hearts. At this stage, the epicardium is properly attached in both groups, though COL4 expression remains lower in *Wt1*^Cre^; *Sox9*^fl/fl^; *R26*^mG^ specimens. Arrowheads in (F’–F”’) highlight the subepicardial expression of COL4, while arrows in (G’–G”’) indicate reduced subepicardial COL4 expression. (Control hearts are from littermate matched *Wt1*^Cre^; *Sox9*^fl/+^; *R26*^mG^ specimens. Endo, endocardium; LV, left ventricle; Myo, myocardium; OFT, outflow tract; RV, right ventricle).

Type IV collagen is the most abundant protein component of basement membranes—a dense ECM layer on the basal surface of epithelia that forms a scaffolding anchoring the epithelium to the underlying tissue or matrix [43]. Morphological assessment of hearts at E12.5 revealed defective attachment of the epicardium to the underlying myocardium in 5/7 of *Wt1*^Cre^; *Sox9*^fl/fl^; *R26*^mG^ specimens, while all control littermates displayed a close apposition of the epicardium to the myocardium (Fig 3A–3C). When examined by immunofluorescence, *Wt1*^Cre^; *Sox9*^fl/fl^; *R26*^mG^ specimens displayed reduced expression of type IV collagen (COL4) in regions of blistering when compared to controls at E12.5 (Fig 3D–E”’). To further evaluate the temporal dynamics of COL4 expression and epicardial integrity, we performed additional immunostaining at E14.5 and E16.5. At E14.5, while subepicardial expression of COL4 remained reduced in *Wt1*^Cre^; *Sox9*^fl/fl^; *R26*^mG^ specimens compared to controls, the epicardium appeared properly attached in all specimens examined (Fig 3F–G”’). This suggests that *Sox9* is not required for maintaining epicardial attachment at this stage. By E16.5, COL4 levels were similarly low in both control and *Wt1*^Cre^; *Sox9*^fl/fl^; *R26*^mG^ hearts (S3 Fig), indicating a broader downregulation of this basement membrane component through development. These findings suggest that *Sox9* may play a temporally specific role during the initial attachment of the epicardium at E12.5, but is dispensable for maintenance of this attachment at later stages. There is supporting evidence that SOX9 activates the *Col4a2* promoter in mouse kidney mesangial cells [44], and that siRNA-mediated knockdown of *SOX9* leads to a reduction in COL4 protein in human mesangial cells [45], although this regulation may be indirect. Taken together, our results support a model in which *Sox9* regulates expression of basement membrane components such as COL4 during early epicardial development, and that disruption of this regulation may contribute to the epicardial blistering observed at E12.5 in *Wt1*^Cre^; *Sox9*^fl/fl^; *R26*^mG^ specimens.

### EPDC invasion correlates with vascular plexus formation

**Fig 4:**
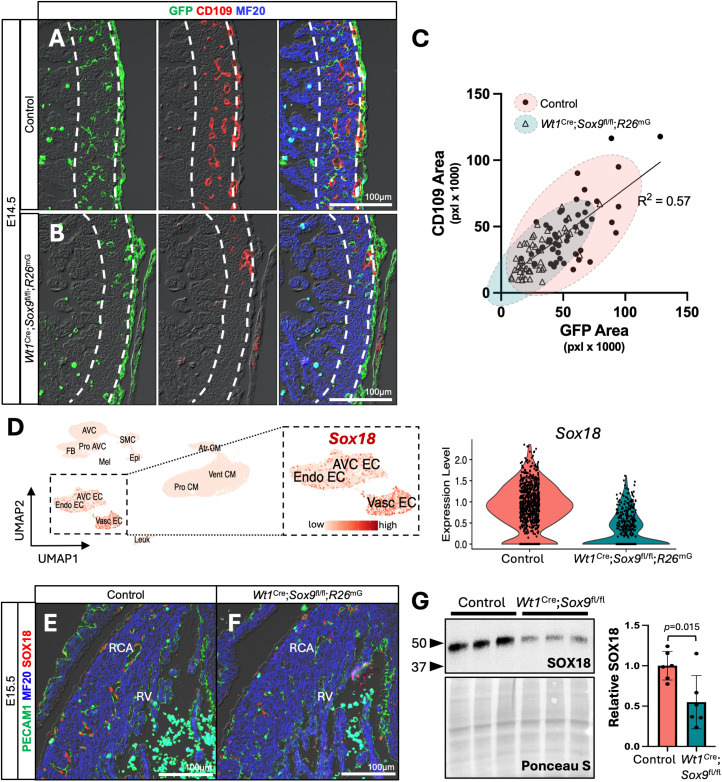
Reduced EPDC invasion correlates with deficient coronary vascular plexus development. **(A, B)** Immunofluorescent stains marking EPDCs (GFP, green), vasculature (CD109, red), and myocardium (MF20, blue) in control (A) and *Wt1*^Cre^; *Sox9*^fl/fl^; *R26*^mG^ (B) hearts at E14.5. Area between dotted lines represents compact ventricular myocardium. **(C)** Linear regression analysis reveals a positive correlation (R^2^ = 0.57) between invading EPDCs and vasculature within the myocardial wall. Datapoints represent quantification from individual image fields, 18 fields per specimen, n = 3 specimens per genotype. 95% confidence ellipses show that sections from control hearts (red ellipse) tend to have more EPDC invasion and more CD109 + vasculature than *Wt1*^Cre^; *Sox9*^fl/fl^; *R26*^mG^ specimens (blue ellipse). **(D)** Magnification of the endothelial portion of the UMAP from Fig 2C with a feature plot of *Sox18* shows that *Sox18* is primarily expressed in the Vasc EC cluster. Violin plot shows *Sox18* expression levels in in control and *Wt1*^Cre^; *Sox9*^fl/fl^; *R26*^mG^ Vasc EC cells. Dots represent relative expression level of individual cells. **(E, F)** Immunofluorescent stains marking vasculature (PECAM1, green), myocardium (MF20, blue), and SOX18 (red) suggest a decrease in SOX18 expression in the vasculature of *Wt1*^Cre^; *Sox9*^fl/fl^; *R26*^mG^ specimens compared to controls at E15.5. **(G)** Western blot confirms downregulation of SOX18 in protein isolated from E15.5 whole hearts (n = 6, 3 independent litters). Data are mean ±s.d. (Student’s unpaired t-test). (Control hearts are from littermate matched *Wt1*^Cre^; *Sox9*^fl/+^; *R26*^mG^ specimens. RCA, right coronary artery; RV, right ventricle).

EPDCs play important roles in the formation of the coronary vascular network, with many mouse models with perturbed epicardial formation and EPDC invasion also displaying significant vascular defects [42,46–51]. Given the proportional decrease in Vasc ECs in *Wt1*^Cre^; *Sox9*^fl/fl^; *R26*^mG^ hearts (S1 Fig) and the downregulation of genes related to vascular development (Fig 2G), we wanted to investigate the relationship between reduced EPDC invasion observed in *Wt1*^Cre^; *Sox9*^fl/fl^; *R26*^mG^ specimens (Fig 1) and the development of the coronary vascular plexus. To do this, we quantified the fluorescent area of EPDCs (GFP) and vasculature as marked by CD109—a cell surface protein we identified previously as marking the coronary vascular plexus [13]—within the compact myocardium of the ventricular walls and performed a linear regression analysis (Fig 4A–4C). Regression analysis revealed a positive correlation of moderate strength between EPDC invasion and CD109 + vasculature (R^2^ = 0.57, Fig 4C). Datapoints from *Wt1*^Cre^; *Sox9*^fl/fl^; *R26*^mG^ tissue sections tended to fall lower along the line of best fit (Fig 4C, red 95% confidence ellipse), indicative of less EPDC invasion and less CD109 + vasculature in *Wt1*^Cre^; *Sox9*^fl/fl^; *R26*^mG^ specimens relative to controls, which fell higher along the line (Fig 4C, blue 95% confidence ellipse). This correlation reinforces a large body of existing research highlighting the importance of the presence of EPDCs in the myocardial wall for proper vascular development.

We next went back to our scRNA-seq data to probe for genes differentially expressed by genotype in the Vasc EC cluster (S2 Table). This revealed a nearly 3.5-fold downregulation of the *Sox18* transcript in *Wt1*^Cre^; *Sox9*^fl/fl^; *R26*^mG^ Vasc ECs compared to controls (Fig 4D). *Sox18* encodes a pro-angiogenic transcription factor that plays cooperative roles with *Sox7* and *Sox17* in regulating angiogenesis [52,53]. Mutations in *Sox18* lead to vascular defects and rupture in the Ragged spontaneous mutant mouse [54–56]. Immunofluorescent labeling for SOX18 (Fig 4E, 4F) and Western blot analysis of protein isolated from control and *Wt1*^Cre^; *Sox9*^fl/fl^; *R26*^mG^ hearts at E15.5 (Fig 4G) confirmed downregulation of SOX18 protein in *Wt1*^Cre^; *Sox9*^fl/fl^; *R26*^mG^ specimens, suggesting a potential role for *Sox18* in the delayed coronary vascular plexus phenotype. Further investigation is required to determine mechanisms by which loss of *Sox9* in the epicardial lineage leads to downregulation of *Sox18* in Vasc ECs, and whether this downregulation directly contributes to delayed coronary plexus formation in *Wt1*^Cre^; *Sox9*^fl/fl^; *R26*^mG^ specimens.

### Epicardial subpopulation enriched in Wt1^Cre^; Sox9^fl/fl^; R26^mG^ hearts

**Fig 5:**
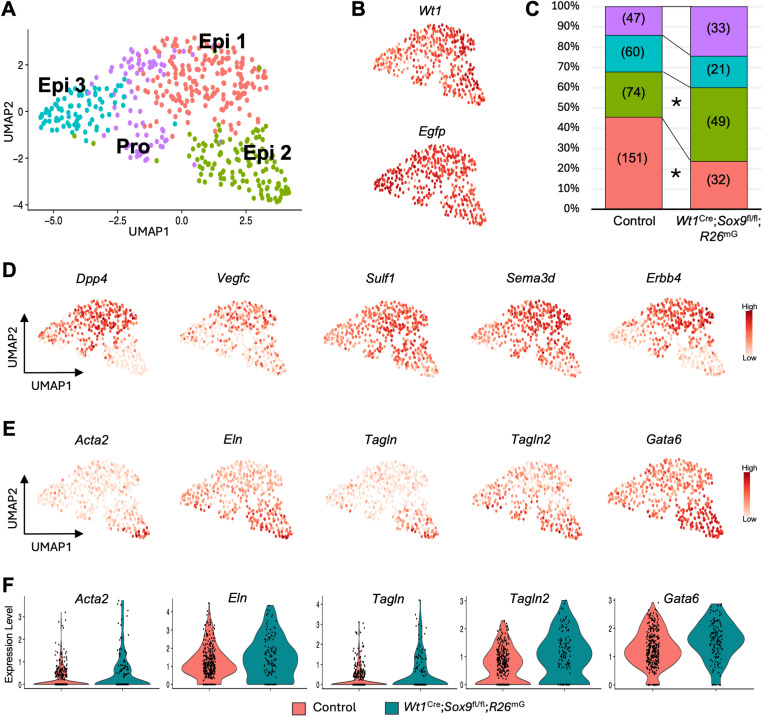
Single-cell transcriptomic profiling of control and *Wt1*^Cre^; *Sox9*^fl/fl^; *R26*^mG^ epicardial cells. **(A)** UMAP depicting a subsetting and reclustering of the epicardial cell cluster. **(B)** Feature plots of epicardial marker *Wt1* and reporter transcript *Egfp* indicated expression in all subpopulations of epicardial cells. **(C)** Relative proportion of cells in each cluster from (A) split by experimental group. Frequencies are listed in parentheses, and asterisks indicate significant differences in frequency between groups (chi-squared analysis, * p < 0.0025). **(D)** Feature plots projected on UMAP from (A) indicating expression of a subset of genes enriched in Epi 1 with red indicating high expression. **(E)** Feature plots projected on UMAP from (A) indicating expression of a subset of genes enriched in Epi 2 with red indicating high expression. **(F)** Violin plots indicating relative expression levels of genes from (E) in control and *Wt1*^Cre^; *Sox9*^fl/fl^; *R26*^mG^ epicardial cells. In all violins, dots represent relative expression level of individual cells.

To investigate transcriptional distinctions between genotypes in more depth, we performed subsetting and reclustering on the Epi cluster (Fig 5A). This revealed four epicardial subpopulations, each expressing epicardial marker *Wt1* and reporter gene *Egfp* (Fig 5B). Chi-square analysis of cell frequencies in each subpopulation revealed a significant enrichment of control cells in the Epi 1 subpopulation, and an enrichment of *Wt1*^Cre^; *Sox9*^fl/fl^; *R26*^mG^ cells in Epi 2.(Fig 5C). When we looked at marker genes expressed in these subpopulations, we saw that Epi 1 is marked by genes with reported roles in coronary vascular development: vascular endothelial growth factor C (*Vegfc*), which promotes the formation of the coronary plexus when secreted by the epicardium [29]; dipeptidyl-peptidase 4 (*Dpp4*), a cell surface protease that cleaves extracellular matrix and matrix-embedded growth factors to enable cell invasion and migration promoting vascularization [57]; Sulfatase 1 (*Sulf1*), a heparan sulfate-editing enzyme promotes cardiac angiogenesis by freeing vascular endothelial growth factors; and semaphoring 3D (*Sema3d*), a marker of cells from the proepicardial organ which contribute to the coronary vascular endothelium [58] (Fig 5D, S3 Table). Interestingly, Epi 1 is also enriched for *Erbb4* (Fig 5D), a neuregulin receptor that regulates cell growth and survival in the myocardium [59] but has not yet been interrogated in the epicardium. Epi 2, which was enriched with *Wt1*^Cre^; *Sox9*^fl/fl^; *R26*^mG^ cells, was characterized by expression of genes often used as markers of VSMC and pericyte differentiation, including alpha-smooth muscle actin (*Acta2*) [60], transgelin 1 and 2 (*Tagln* and *Tagln2*) [61–63], elastin (*Eln*) [64,65], and GATA-binding 6 (*Gata6*) [66,67] (Fig 5E, S3 Table). These genes not only marked the Epi 2 cluster but were expressed in higher levels in *Wt1*^Cre^; *Sox9*^fl/fl^; *R26*^mG^ epicardial cells compared to controls (Fig 5F). Epi 3 was marked by high levels of ribosomal genes indicative of cellular stress, while the Pro Epi cluster expressed high levels of proliferation genes including *Mki67, Cenpa, and Top2a* (S3 Table).

To further characterize these subpopulations, we looked at the epicardial-derived fibroblast marker Tcf21 and determined this was not enriched in any specific subpopulation of the epicardium (S2 Fig), although Tcf21 was significantly downregulated in Wt1^Cre^; Sox9^fl/fl^; R26^mG^ epicardium and fibroblast clusters compared to controls (Fig 3D, S4 Fig, S2 Table). Nor were the EMT markers Snai1 or Snai2 enriched in any subpopulation of epicardial cells (S2 Fig), suggesting that Epi 2 is not a cluster undergoing EpiMT, but may represent a cluster of epicardial cells prematurely differentiating into VSMCs, or primed for the mural cell fate. Interestingly, Tbx18 expression was notably weak in this subpopulation (S2 Fig), which has been reported to have an inhibitory effect on VSMC differentiation [68,69]. These observations are supported by prior literature independently describing roles for Sox9, Tcf21, and Tbx18 in the repression of SMC differentiation [38,68–73], such that loss of Sox9 and subsequent downregulation of Tcf21 in Wt1^Cre^; Sox9^fl/fl^; R26^mG^ hearts leads to enrichment of a Tbx18-low epicardial subpopulation expressing high levels of VSMC markers. An important limitation of these data to note is that the E14.5 timepoint is before coronary VSMCs have matured, making further investigation of later timepoints critical for confirming a role for Sox9 in epicardial-derived VSMC fate determination. Loss of Sox9 alters the spatial distribution of EPDCs.

### Loss of *Sox9* results in depletion of interstitial EPDCs

**Fig 6:**
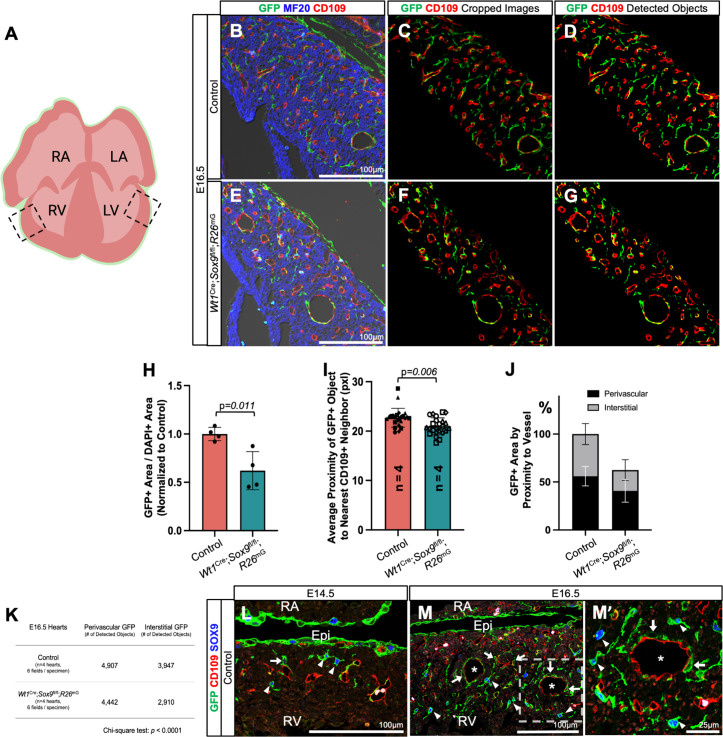
Altered spatial distribution of EPDCs in *Wt1*^Cre^; *Sox9*^fl/fl^; *R26*^mG^ hearts. **(A)** Schematic depicting the ventricular regions analyzed (boxed). **(B–G)** Proximity analysis workflow. Immunofluorescent staining of E16.5 heart sections from control (B) and *Wt1*^Cre^; *Sox9*^fl/fl^; *R26*^mG^ (E) hearts showing EPDCs (GFP, green), coronary vasculature (CD109, red), and myocardium (MF20, blue). (C) and (F) are representative images used for analysis, showing GFP and CD109 channels cropped to remove the epicardium proper, trabeculated myocardium, and ventricular lumen. (D) and (G) show the GFP+ (green) and CD109+ (red) objects detected by Otsu’s auto-thresholding method. **(H)** Quantification of GFP+ fluorescence area, first normalized to DAPI area to account for myocardial area differences, then normalized to control (set to 1.0) for comparison between genotypes (n = 4, Student’s unpaired t-test). **(I)** Nearest-neighbor analysis reveals a decreased average distance between GFP+ object centroids and their nearest CD109 + neighbor in *Wt1*^Cre^; *Sox9*^fl/fl^; *R26*^mG^ specimens. Each datapoint is the average distance to the nearest CD109 + neighbor for each GFP+ object in a single image. Datapoint shapes correspond to technical replicates (images) for each specimen (n = 4 specimens, 6 images per specimen, Student’s unpaired t-test). **(J)** Proportion of GFP+ fluorescence area classified as perivascular (black) or interstitial (gray), based on proximity to nearest CD109 + neighbor, as described in Materials and Methods. **(K)** Table summarizing object detection counts for perivascular and interstitial GFP+ objects by genotype. Chi-square analysis indicates a significant shift in spatial distribution (p < 0.0001). (L–M’) Immunofluorescence shows SOX9 expression (blue), primarily in EPDCs (GFP, green) positioned interstitially (arrowheads), while those positioned near vasculature (CD109, red), are largely SOX9-negative (arrows). Data are mean ± s.d. (Control hearts are from littermate matched *Wt1*^Cre^; *Sox9*^fl/+^; *R26*^mG^ specimens. LA, left atrium; LV, left ventricle; RA, right atrium; RV, right ventricle).

It is notoriously difficult to distinguish vascular smooth muscle cells (VSMCs) from fibroblasts using transcriptional markers, as many commonly used genes are expressed in both lineages [74]. To investigate whether loss of *Sox9* preferentially affects one EPDC lineage over the other, we used a proximity-based approach to assess the spatial relationship between GFP+ EPDCs and the coronary vasculature. We reasoned that EPDCs in close proximity to coronary vasculature are more likely to be mural cells (VSMCs and pericytes), while those located further from vasculature are more likely to be fibroblasts. At E14.5, VSMC coverage of the developing coronary plexus is patchy and incomplete, as demonstrated by Volz et al. [75], so we analyzed ventricular sections at E16.5 (Fig 6A–6G). This reflects a timepoint at which coronary arteries have begun to mature and mural cells have been recruited to the vessel wall. We performed immunofluorescent staining for EPDCs (GFP), coronary endothelial cells (CD109), and myocardium (MF20), and we generated image analysis code to detect GFP+ and CD109 + objects in cropped regions of compact myocardium (Fig 6B–6G).

Fluorescence quantifications revealed that the overall GFP+ area was significantly reduced in *Wt1*^Cre^; *Sox9*^fl/fl^; *R26*^mG^ hearts compared to controls, confirming a sustained reduction in EPDC invasion at later stages (Fig 6H). To explore whether this reduction reflected a shift in EPDC localization, we measured the average distance from each GFP+ cell to its nearest CD109 + neighbor. In *Wt1*^Cre^; *Sox9*^fl/fl^; *R26*^mG^ hearts, GFP+ cells were significantly closer to the vasculature than in controls (Fig 6I), suggesting that loss of *Sox9* alters the spatial distribution of EPDCs within the myocardium. To better understand this shift, we binned GFP+ cells into “perivascular” or “interstitial” groups based on their proximity to the nearest CD109 + cell. This analysis revealed that the reduction in GFP+ area in mutants was primarily due to loss of interstitial EPDCs, with perivascular EPDCs remaining relatively unaffected (Fig 6J). Object counts confirmed a significant redistribution of EPDCs in the mutant hearts (Fig 6K).

Lastly, we examined SOX9 protein expression at E14.5 and E16.5, which confirmed that SOX9 was primarily expressed in interstitial EPDCs (Fig 6L–M’, arrowheads) and largely absent from perivascular cells (Fig 6L–M’, arrows). These findings suggest that SOX9 is important for the maintenance of the epicardial-derived fibroblast lineage, and that its loss results in a selective depletion of this population.

## Discussion

This report provides insight into the roles of *Sox9* in the epicardium as it relates to the regulation of migratory and differentiation events important for proper heart development. Epicardial-specific loss of *Sox9* led to a delay in the attachment of the epicardium to the myocardium, as evidenced by a high prevalence of epicardial blistering or blebbing away from the myocardium in E12.5 *Wt1*^Cre^; *Sox9*^fl/fl^; *R26*^mG^ specimens. While this blistering is no longer observed at E14.5, reduced invasion in *Wt1*^Cre^; *Sox9*^fl/fl^; *R26*^mG^ points to an important role for *Sox9* in regulating the proper attachment and spatiotemporally regulated invasion of EPDCs into the myocardium.

Our single-cell transcriptomic analysis identified key regulators of epicardial development that were differentially expressed in *Wt1*^Cre^; *Sox9*^fl/fl^; *R26*^mG^ specimens compared to controls at E14.5. Notably, loss of *Sox9* led to downregulation of two transcription factors reported to influence epicardial heterogeneity and differentiation potential, *Bnc1* [39] and *Tcf21* [34,37,38], as well as dysregulation of factors influencing EpiMT including *Tcf21* [34], *Cebpb* and *Cebpd* [33], and *Crip1* [36]. In human pluripotent stem cell-derived epicardium (hPSC-epi), BNC1 and TCF21 marked distinct subpopulations, with TCF21 + hPSC-epi cells retaining the ability to differentiate into both fibroblasts and VSMCs, while BNC1 + hPSC-epi cells predominantly differentiated into VSMCs [39]. While these subpopulations were not specifically observed in our dataset, downregulation of both *Bnc1* and *Tcf21* in *Wt1*^Cre^; *Sox9*^fl/fl^; *R26*^mG^ epicardial cells is suggestive of an epicardium with diminished differentiation capacity. In mice, *Tcf21* represses transcription of VSMC genes, and regulates lineage-specific EpiMT of cardiac fibroblasts [34,73]. Additional regulators of EpiMT include C/EBP-family transcription factors, which promote *Wt1* and *Raldh2* expression in the epicardium, and are reactivated after ischemic injury [33]. Additionally, lower expression of basement membrane collagens and growth factors secreted by the epicardium provide potential explanations for the epicardial blistering and myocardial hypoproliferation phenotypes observed in *Wt1*^Cre^; *Sox9*^fl/fl^; *R26*^mG^ specimens.

We observed a correlation between EPDC invasion and the presence of the coronary vascular plexus in this model, where reduced EPDCs in the myocardium of *Wt1*^Cre^; *Sox9*^fl/fl^; *R26*^mG^ specimens correlates with reduction in CD109-expressing vasculature within the myocardial wall. This observation bolsters a large body of literature describing the importance of EPDCs in the proper development and patterning of the coronary vascular plexus [31,42,69,76,77]. We identified the pro-angiogenic transcription factor SOX18 [52–54] as a potential mediator of this phenotype, though the mechanisms of how signaling from EPDCs may influence SOX18 expression in Vasc ECs remains unclear.

We discovered in *Wt1*^Cre^; *Sox9*^fl/fl^; *R26*^mG^ hearts an expansion of a subpopulation of epicardial cells with characteristics of both epicardial cells and differentiating VSMCs (Epi 2), and a corresponding reduction of epicardial cells marked by expression of proangiogenic factors (Epi 1). Previous literature has implicated *Sox9* in playing an inhibitory role in VSMC differentiation by suppressing the transcription of VSMC- specific gene programs (*Acta2*, *Cnn1*, *Tagln*) through its interaction with myocardin (MYOCD) [70,71]. Repression of *Sox9* by the canonical Notch ligand Jagged1 (JAG1) has also been reported to be important for VSMC differentiation, as the lack of this repression, or the misexpression of *Sox9*, resulted in a differentiation of VSMC precursors into chondrocytes in the developing aorta [72]. Additional roles for *Tcf21* and *Tbx18* in inhibiting or repressing VSMC differentiation have also been described previously: epicardial loss of *Tcf21* or epicardial expression of an transcriptional activator form of *Tbx18* (instead of its wildtype transcriptional repressor activity [78]) each result in premature differentiation of epicardial cells into VSMCs [38,69,73]. The studies alongside our transcriptomic observations support a model in which the loss of *Sox9* and subsequent downregulation of *Tcf21* in *Wt1*^Cre^; *Sox9*^fl/fl^; *R26*^mG^ epicardium leads to expansion of a *Tbx18*-low subpopulation of epicardial cells expressing higher levels of VSMC genes.

An unbiased proximity-based image analysis approach to interrogate the spatial relationship between EPDCs and coronary vasculature supported our hypothesis that loss of *Sox9* is preferentially detrimental to the interstitial mesenchymal EPDC populations, while leaving the perivascular EPDC populations intact. Future studies can incorporate additional timepoints to further investigate maturation and differentiation trajectories at a transcriptional level and dissect how loss of Sox9 influences these trajectories.

**Fig 7:**
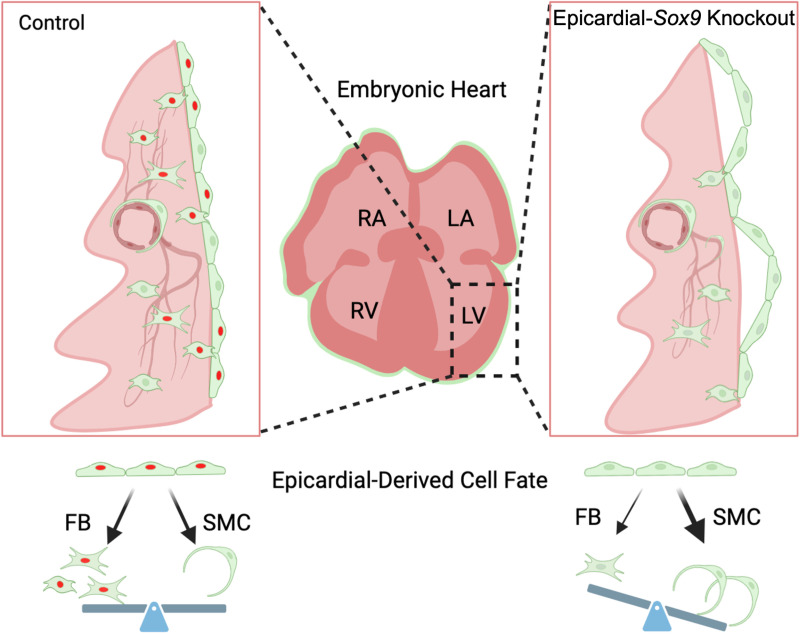
Model for roles of *Sox9* in epicardial development. Analyses of *Wt1*^Cre^; *Sox9*^fl/fl^; *R26*^mG^ hearts reveal roles for *Sox9* in epicardial attachment, invasion, and differentiation important for the proper development of the myocardium and coronary vascular plexus. Transcriptomic analyses suggest a lineage preference towards the VSMC fate in response to loss of *Sox9*.

Together, these data identify an important role for *Sox9* in epicardial attachment, invasion, and differentiation that is important for proper myocardial and coronary vascular development. Fig 7 is a model illustrating the phenotypic consequences of loss of *Sox9* in the epicardial lineage. *Sox9* deletion leads to aberrant epicardial attachment, reduced EPDC invasion, and a preference towards the VSMC fate. Reduced expression of *Tcf21* in *Wt1*^Cre^; *Sox9*^fl/fl^; *R26*^mG^ specimens may mediate lineage specific effects of *Sox9* deletion, and reduced expression of *Sox18* may contribute to a deficiency in coronary plexus development, but further analyses are required to confirm mechanisms underlying these phenotypes.

Understanding how epicardial cells function to regulate the growth and development of the myocardium and coronary vasculature has intriguing therapeutic implications. Existing evidence supports that the “reactivation” or “reprogramming” of the adult epicardium to a developmental state may promote revascularization and improve ejection fraction after ischemic injury to the myocardium, though there are significant limitations preventing translation of these findings into clinical practice currently [33,79–83]. These include limitations in how we understand the molecular initiation and termination of these activation signals, as well as the potential for functional divergence between the human epicardial response when compared to model organisms such as mouse. Further research into mechanisms of epicardial invasion, differentiation, and function could illuminate novel approaches to improve cardiac outcomes in patients with congenital heart disease, diseases of the coronary system, or ischemic injury.

## Materials and methods

### Animals

All animal experiments were conducted according to NIH guidelines (Guide for the Care and Use of Laboratory Animals) and protocols approved by the MUSC Institutional Animal Care and Use Committee (IACUC) with protocol ID number IACUC-2020-01140. The mWt1/IRES/GFP-Cre (*Wt1*^Cre^) mouse was described previously [11]. The B6.129S7-Sox9^tm2Crm^/J (Sox9^fl/fl^) mouse and the B6.129(Cg)-Gt(Rosa)26Sor^tm4(ACTB-tdTomato,EGFPLuo^/J (*Rosa26*^mTmG^ dual fluorescence lineage trace reporter expressing membrane bound tdTomato or eGFP, abbreviated *R26*^mG^) mouse was obtained from the Jackson Laboratory. Epicardial-specific *Sox9* knockout animals (*Wt1*^cre^; *Sox9*^fl/fl^; *R26*^mG^) and littermate control (*Wt1*^cre^; *Sox9*^fl/+^; *R26*^mG^ or *Wt1*^cre^; *Sox9*^+/+^; *R26*^mG^) animals were generated by crossing *Wt1*^cre^; *Sox9*^fl/+^ males with *Sox9*^*f*l/+^; *R26*^mG^ or *Sox9*^fl/fl^; *R26*^mG^ females. Mice heterozygous at the *Sox9* locus (*Wt1*^cre^; *Sox9*^fl/+^; *R26*^mG^) did not present with any of the phenotypes described in *Wt1*^cre^; *Sox9*^fl/fl^; *R26*^mG^ animals and were thus deemed appropriate controls for histological, immunofluorescence, and transcriptomic analyses. Pregnant dams were euthanized by isoflurane induction followed by cervical dislocation in accordance with NIH guidelines. Embryos were considered day 0.5 at midday on the day of vaginal plug detection, and staging was confirmed upon isolation using distinctive features described in *The House Mouse: Atlas of Embryonic Development* [84]. Both male and female specimens were generated, and combined data for both sexes are shown.

### Histology, immunofluorescence, and microscopy

Embryos were dissected in 1x PBS and fixed in 4% paraformaldehyde (PFA) either for 4 hours at room temperature or overnight at 4°C. Embryos were then processed through a series of graded alcohols, cleared in toluene, and embedded in paraffin. Hematoxylin and Eosin (H&E) or immunofluorescence staining was performed on 5µm tissue sections. Slides were deparaffinized in xylenes and rehydrated through graded alcohols. Antigen retrieval was performed by pressure cooking slides in citric acid-based antigen unmasking solution (Vector, H3300) for 1 min. Sections were incubated for 30 mins with 1% bovine serum albumin (BSA) prior to immunostaining to minimize non-specific binding of primary antibodies. Sections were incubated in primary antibody overnight at 4°C, washed in 1x PBS, and incubated with secondary antibody for 1 hour at room temperature. Serial dilutions were performed to determine optimal antibody concentrations, and secondary only controls were used to confirm signal specificity for the primary antibody. Primary and secondary antibodies and dilutions can be found in S4 Table. Slides were coverslipped with SlowFade Gold Antifade Reagent with DAPI (Invitrogen). Brightfield images were acquired using Olympus BX40. Fluorescence images were acquired with Zeiss AxioImager II and Leica TCS SP8 microscopes.

### Epicardial explant culture and scratch assay

Epicardial explant culture was carried out essentially as previously described [85]. Briefly, Falcon® 4-well culture slides (Corning 354114) were precoated with rat tail collagen (Corning CB-40236), coated with media (M199 + 10% Fetal Bovine Serum), and warmed in incubator for 30 mins, before removing media. Hearts were microdissected from E12.5 embryos, placed dorsal side down in individual chambers, and incubated at 37°C, 5% CO_2_ for 45 mins to allow adherence to slide. After 45 mins, 200uL of media was added to each chamber, and slides were incubated overnight at 37°C, 5% CO_2_. The next day, hearts were carefully removed and media was changed. After removal of the heart, explant cultures were incubated for 24 hours to allow for closure of acellular area where heart was attached to the plate. The next day, after confirming confluency of explant culture, cultures were scratched with a p10 pipette tip to create acellular area and imaged at t = 0,8,12, and 16 hours post scratch on a Zeiss AxioImager II microscope. Remaining acellular area was quantified in Adobe Photoshop and plotted by genotype in GraphPad Prism 9.

### Single-cell RNA-sequencing

Embryos were dissected at E14.5, tail clipped, and placed in 2% BSA in ice cold PBS during genotyping with the KAPA Fast Genotyping Kit (~2 hours). Hearts were then pooled by genotype, dissected and dissociated in 200ug/mL Liberase TM (Sigma-Aldrich) dissolved in Hibernate E minus calcium media (Transnetyx). Single-cell suspensions loaded onto a 10X Genomics Next GEM Chip and emulsified with 3’ Single Cell Next GEM beads using a ChromiumTM Controller (10X Genomics). All embryos were dissected, digested, and barcoded on the same day to minimize variation. From barcoded cDNAs, gene expression libraries were constructed using ChromiumTM Single Cell 3′ Library Kits (10X Genomics) at the Translational Science Laboratory (Medical University of South Carolina). Next-generation sequencing was performed on each sample using an Illumina NovaSeq S4 flow cell at the VANTAGE facility (Vanderbilt University Medical Center). Fastq files were aligned to the mm10 reference transcriptome using the count function in the Cell Ranger software (v7.1, 10x Genomics, Pleasanton, CA, USA). Quality control was performed using a pipeline including exclusion of cells containing high mitochondrial read fraction, low detection of unique genes/ number of unique molecular identifiers per cell, and doublet filtering. Dimensional reduction and unsupervised clustering were performed RunUMAP and FindClusters functions in Seurat. Differential expression analyses were carried out in Seurat using DESeq2. Visualization and graphics were generated in Ggplot2. Functional analyses were conducted using ToppFun [32].

### Western blot

Protein was harvested from dissected embryonic hearts using 1X RIPA buffer (Thermo, 89900) with 1X Protease Inhibitor Cocktail (Abcam, ab271306), boiled at 95 °C for 5 mins with 4X Laemmli Buffer (BioRad, 1610747) with 10% 2-mercaptoethanol, and separated by gel electrophoresis using a BioRad mini blot system with 4−20% Mini PROTEAN TGX Stain-Free Protein Gels (BioRad, 456-8093). Protein was transferred to a nitrocellulose membrane using a BioRad Trans-Blot Turbo Semi-Dry Transfer System with Trans-Blot Turbo Mini Nitrocellulose Transfer Packs (BioRad, 170-4158). Membranes were washed in Ponceau S total protein stain (Thermo, A40000478) and imaged on a BioRad ChemiDoc MP imaging system for downstream normalization. Membranes were washed in Tris Buffered Saline with 1% Tween-20 (TBST) to remove Ponceau S, then blocked with 5% skim milk diluted in TBST. Membranes were then incubated overnight at 4 °C in primary antibody diluted 1:1000 in blocking buffer. Following three 10-min washes in TBST, membranes were then incubated at room temperature for 1 hour in HRP-conjugated secondary antibody diluted 1:7500 in blocking buffer. Following three 10-min washes in TBST, membranes were exposed to Pierce ECL Western Blotting Substrate (ThermoFisher, 32209) and imaged on a BioRad ChemiDoc MP imaging system.

### Proximity-based image analysis

To assess the spatial relationship between epicardial-derived cells (marked by GFP) and coronary vasculature (marked by CD109), a custom image analysis pipeline was developed in R (source code available at https://github.com/andrewbharvey/proximity-based-image-analysis). Images were acquired from n = 4 embryos per genotype with 6 images analyzed per specimen. For each image, regions of interest were cropped to include only the compact myocardium, excluding the epicardium proper and trabecular myocardium. Channels were segmented and GFP+ and CD109 + objects identified by Otsu thresholding. For each GFP+ cell, the shortest distance to the nearest CD109 + neighbor was calculated. Distances were then used to categorize GFP+ cells into bins based on proximity. A threshold of 22 pixels derived from the mean distance between GFP+ and CD109 + cells in control specimens was applied to stratify cells as either “perivascular” (<22 pixels) or “interstitial” (≥22 pixels). This binning enabled quantification of how loss of *Sox9* influences spatial proximity to vasculature as a proxy for determining whether loss of *Sox9* preferentially affects mural cells (perivascular) or interstitial fibroblasts (interstitial). Measurements were analyzed and visualized using GraphPad Prism 9.

### Statistic and quantifications

Statistical analyses were performed in GraphPad Prism 9. Sample sizes and statistical tests are labeled in figure captions. For differential expression analyses of scRNA-seq data, the Wald test was used with Benjamini Hochberg False Discovery Rate adjustment. Genes were considered significant which had a P_adj_ < 0.05 and a |Fold Change| > 1.25. Chi-squared analysis was used to compare relative proportions of cell numbers within epicardial clusters between experimental groups (α = 0.0025). Fisher’s Exact Test was used for analysis of epicardial attachment in n = 7 specimens per genotype (α = 0.05). Simple linear regression analysis was used to analyze correlation between EPDC invasion (GFP+ fluorescent area) and presence of vasculature (CD109 + fluorescent area) in cropped images of ventricular wall, with R^2^ values of 0.75, 0.50, and 0.25 described as substantial, moderate, and weak correlations, respectively, as per Henseler, 2009 [86]. Fluorescence area of GFP, CD109, and DAPI were calculated in CellProfiler 3.1.8. For these measurements, n = 3 specimens per genotype were analyzed, 4 sections (spaced at least 50µm apart) were analyzed per specimen, and 6 fields of view spaced equally around the left and right ventricles were analyzed per section. All data was analyzed and visualized in GraphPad Prism 9. For Western blot analysis of SOX18 levels, n = 6 specimens per genotype were analyzed from 3 independent E15.5 litters. For relative quantification, band intensities were determined in Adobe Photoshop, normalized to total protein intensity from Ponceau S, and compared in GraphPad Prism 9 (Student’s unpaired t-test, α = 0.05).

## Supporting information

S1 Fig Single-cell RNA-sequencing cellular composition. (A) Uniform Manifold Approximation Projection (UMAP) representation of unbiased clustering analysis of 21,647 single-cell transcriptomes identifying 17 transcriptionally distinct clusters (from Fig 2C) (B) Frequency plot depicting the proportional distribution of 21,647 cells across all groups. (D) Feature plots indicating expression of select marker genes projected on UMAP with red indicating high expression.
(TIF)

S2 Fig Transcriptional characteristics of epicardial cells. (A) Violin plots of negative regulators of epithelial-to-mesenchymal transformation *Cldn3* and *Crip1*, which were upregulated in *Wt1*^Cre^; *Sox9*^fl/fl^; *R26*^mG^ epicardial cells compared to controls. (B) Violin plots of secreted cardiomyocyte mitogens *Fgf2*, *Fgf9*, and *Wnt5a*, which were downregulated in *Wt1*^Cre^; *Sox9*^fl/fl^; *R26*^mG^ epicardial cells compared to controls. (C) (D) Feature plot indicated that epicardial-derived fibroblast marker *Tcf21* was not enriched in any subpopulation of epicardial cells. (E) Feature plots of epithelial-to-mesenchymal transformation markers *Snai1* and *Snai2* indicated no enrichment in any subpopulation of epicardial cells. (F) Feature plot of *Tbx18*, a negative regulator of VSMC differentiation, indicated a low-expressing population corresponding with the Epi 2 epicardial subpopulation.
(TIF)

S3 Fig COL4 expression in the epicardium at E16.5. Immunofluorescent staining of ventricular region for GFP (epicardium, green) and type IV collagen (COL4, red) in control (A,B) and *Wt1*^Cre^; *Sox9*^fl/fl^; *R26*^mG^ (C,D) embryonic hearts at E16.5. Merged images (A,C) and COL4 channel only (B,D) demonstrate that subepicardial COL4 expression is low in both conditions at this stage, suggesting a broader developmental downregulation of this basement membrane component. Control hearts are from littermate-matched *Wt1*^Cre^; *Sox9*^fl/+^; *R26*^mG^ specimens.
(TIF)

S4 Fig Dysregulation of genes involved in growth and differentiation in fibroblasts. (A) Violin plots show downregulated expression of *Tcf21* and *Sox9* in *Wt1*^Cre^; *Sox9*^fl/fl^; *R26*^mG^ fibroblasts. Notably, the *Sox9* mRNA transcript is still present in *Wt1*^Cre^; *Sox9*^fl/fl^; *R26*^mG^ specimens but does not make a functional protein product due to Cre-mediated excision of exons 2 and 3. Biological Processes from functional enrichment analysis of significantly downregulated genes (p_adj_ < 0.05, |FC| > 1.25) in *Wt1*^Cre^; *Sox9*^fl/fl^; *R26*^mG^ fibroblasts compared to controls.
(TIF)

S5 Fig Raw blot images. Original blot images from Fig 4G. All images were captured on BioRad ChemiDoc MP imaging system. Lanes not included on cropped figure images are indicated with a red X.
(TIF)

Table S1 E14.5 cluster markers. (XLSX)

Table S2 E14.5 differential expression analyses. (XLSX)

Table S3 Epicardial subsetting markers. (XLSX)

Table S4 Antibodies. (XLSX)
