# Supplementary figures and images for: Sox9 in the epicardium: Implications for cell invasion, differentiation, and coronary vascular development
Source: PLoS One. 2025 Jun 23;20(6):e0325852. doi: 10.1371/journal.pone.0325852 (PMC12185001; doi:10.1371/journal.pone.0325852)

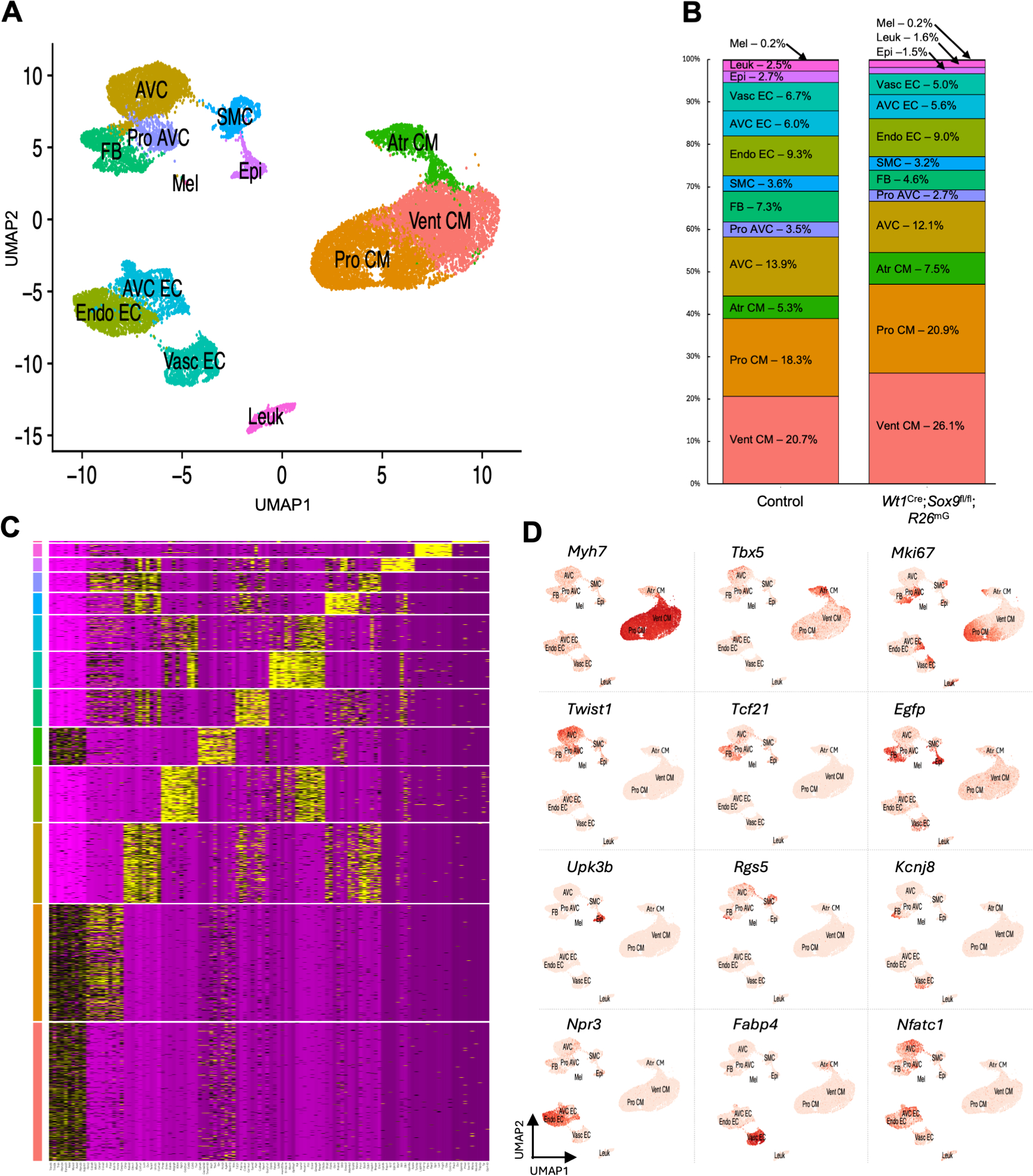

Supplement: S1 Fig — (A) Uniform Manifold Approximation Projection (UMAP) representation of unbiased clustering analysis of 21,647 single-cell transcriptomes identifying 17 transcriptionally distinct clusters (from Fig 2C) (B) Frequency plot depicting the proportional distribution of 21,647 cells across all groups. (D) Feature plots indicating expression of select marker genes projected on UMAP with red indicating high expression. (TIF) [file pone.0325852.s001.tif]

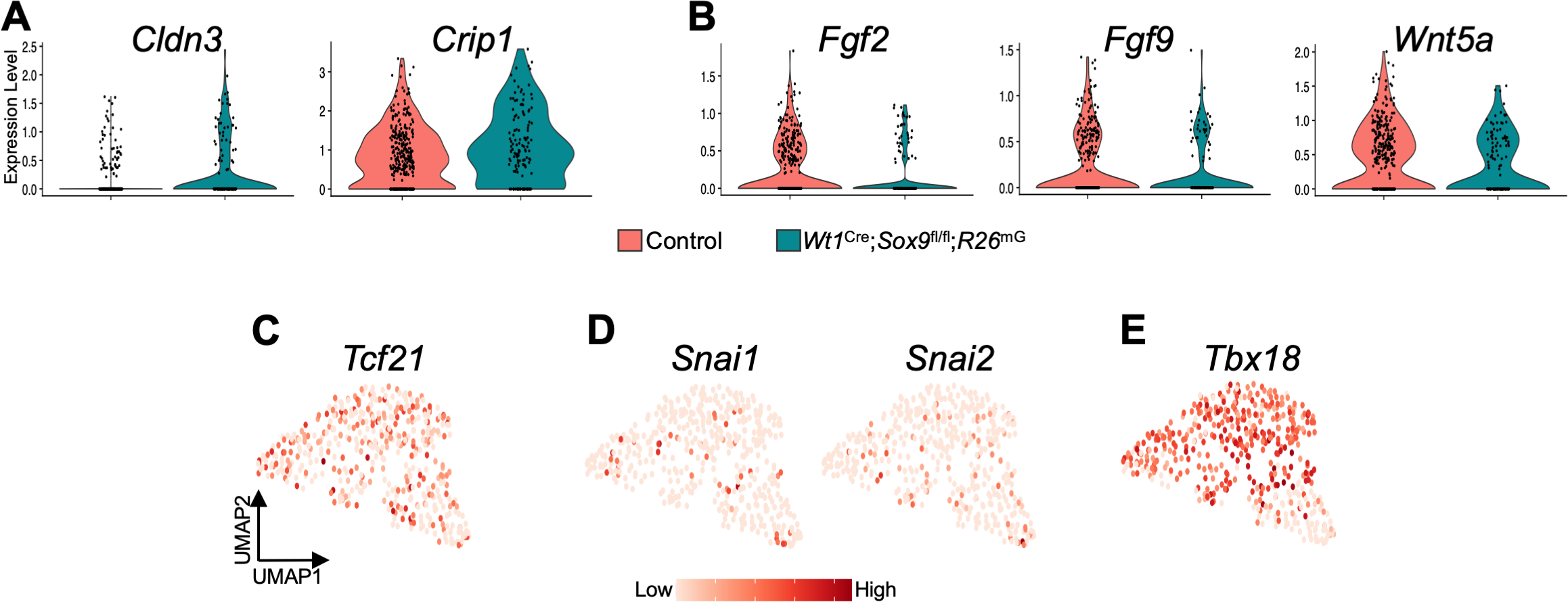

Supplement: S2 Fig — (A) Violin plots of negative regulators of epithelial-to-mesenchymal transformation Cldn3 and Crip1, which were upregulated in Wt1Cre; Sox9fl/fl; R26mG epicardial cells compared to controls. (B) Violin plots of secreted cardiomyocyte mitogens Fgf2, Fgf9, and Wnt5a, which were downregulated in Wt1Cre; Sox9fl/fl; R26mG epicardial cells compared to controls. (C) (D) Feature plot indicated that epicardial-derived fibroblast marker Tcf21 was not enriched in any subpopulation of epicardial cells. (E) Feature plots of epithelial-to-mesenchymal transformation markers Snai1 and Snai2 indicated no enrichment in any subpopulation of epicardial cells. (F) Feature plot of Tbx18, a negative regulator of VSMC differentiation, indicated a low-expressing population corresponding with the Epi 2 epicardial subpopulation. (TIF) [file pone.0325852.s002.tif]

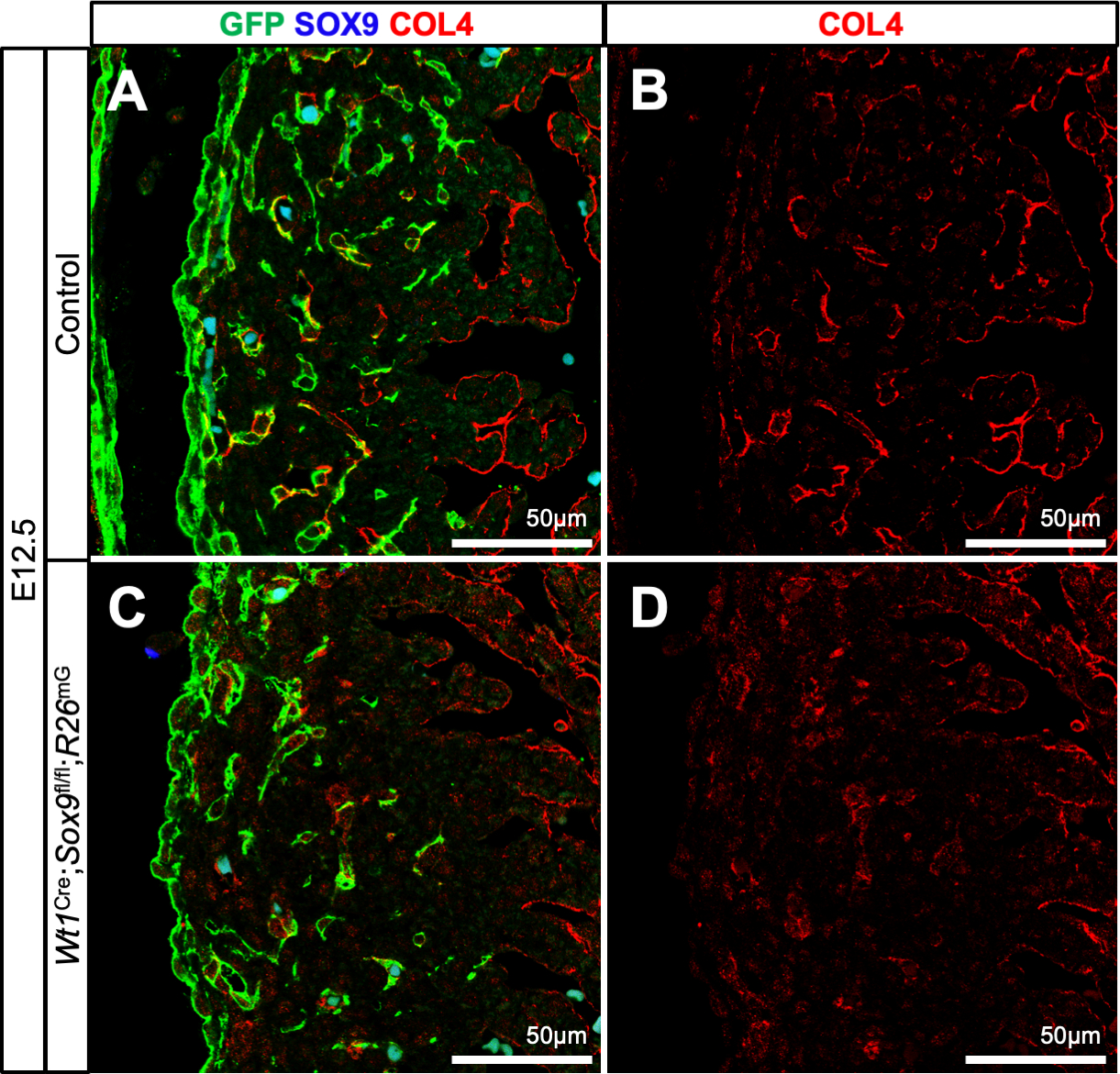

Supplement: S3 Fig — Immunofluorescent staining of ventricular region for GFP (epicardium, green) and type IV collagen (COL4, red) in control (A,B) and Wt1Cre; Sox9fl/fl; R26mG (C,D) embryonic hearts at E16.5. Merged images (A,C) and COL4 channel only (B,D) demonstrate that subepicardial COL4 expression is low in both conditions at this stage, suggesting a broader developmental downregulation of this basement membrane component. Control hearts are from littermate-matched Wt1Cre; Sox9fl/+; R26mG specimens. (TIF) [file pone.0325852.s003.tif]

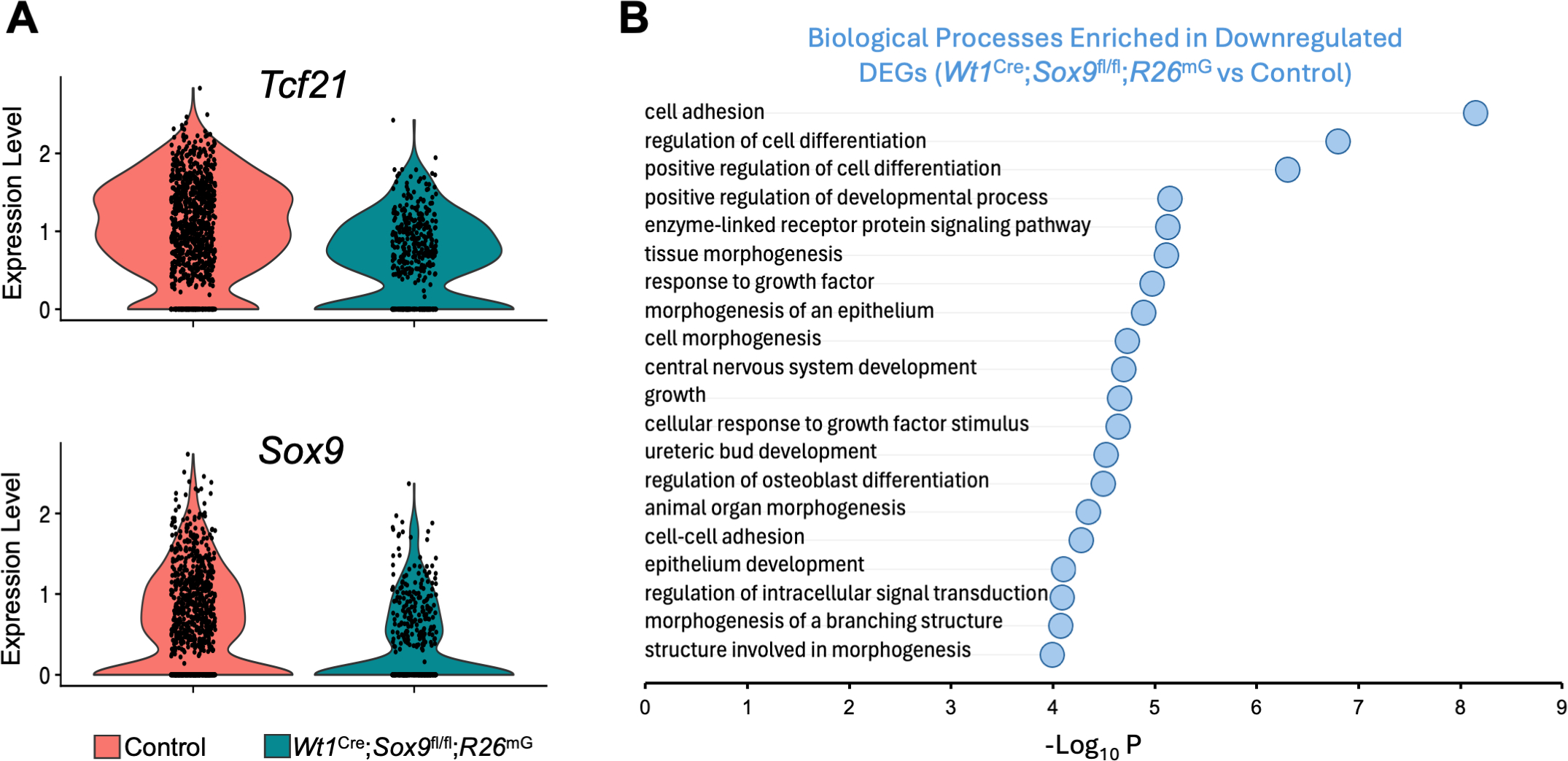

Supplement: S4 Fig — (A) Violin plots show downregulated expression of Tcf21 and Sox9 in Wt1Cre; Sox9fl/fl; R26mG fibroblasts. Notably, the Sox9 mRNA transcript is still present in Wt1Cre; Sox9fl/fl; R26mG specimens but does not make a functional protein product due to Cre-mediated excision of exons 2 and 3. Biological Processes from functional enrichment analysis of significantly downregulated genes (padj < 0.05, |FC| > 1.25) in Wt1Cre; Sox9fl/fl; R26mG fibroblasts compared to controls. (TIF) [file pone.0325852.s004.tif]

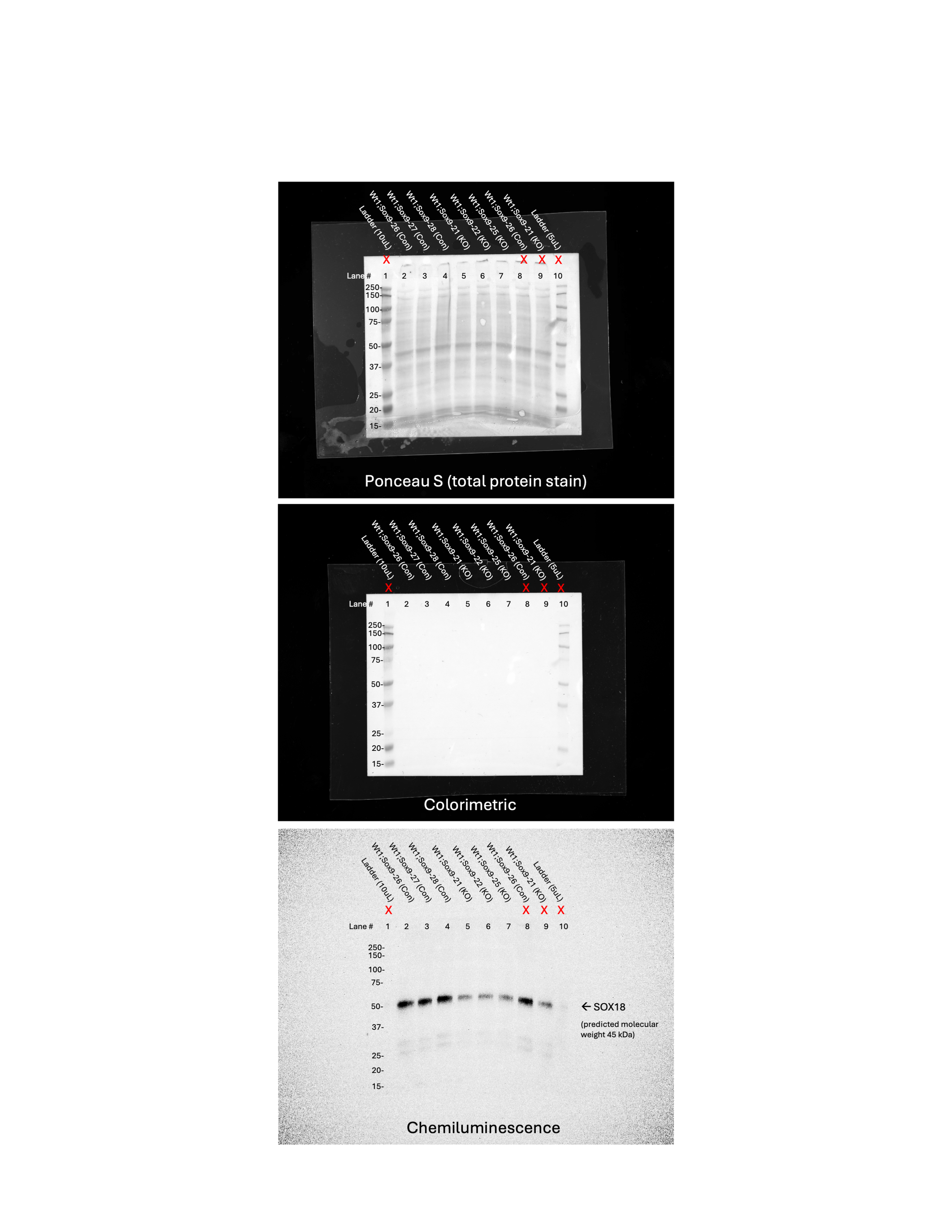

Supplement: S5 Fig — Original blot images from Fig 4G. All images were captured on BioRad ChemiDoc MP imaging system. Lanes not included on cropped figure images are indicated with a red X. (TIF) [file pone.0325852.s005.tif]
